# Predictors of weight loss in patients with obesity treated with a Very Low-Calorie Ketogenic Diet

**DOI:** 10.3389/fnut.2023.1058364

**Published:** 2023-01-25

**Authors:** Ilaria Ernesti, Francesco Baratta, Mikiko Watanabe, Renata Risi, Elisabetta Camajani, Agnese Persichetti, Dario Tuccinardi, Stefania Mariani, Carla Lubrano, Alfredo Genco, Giovanni Spera, Lucio Gnessi, Sabrina Basciani

**Affiliations:** ^1^Surgical Endoscopy Unit, Department of Surgical Sciences, Sapienza University of Rome, Rome, Italy; ^2^Department of Clinical Internal, Anesthesiological and Cardiovascular Sciences, Sapienza University of Rome, Rome, Italy; ^3^Section of Medical Pathophysiology, Food Science and Endocrinology, Department of Experimental Medicine, Sapienza University of Rome, Rome, Italy; ^4^Department of Human Sciences and Promotion of the Quality of Life, San Raffaele Open University, Rome, Italy; ^5^Department of Endocrinology and Diabetes, University Campus Bio-Medico of Rome, Rome, Italy

**Keywords:** fibroblast growth factor 21, insulin resistance, body composition, low carbohydrate diet (LCD), very low energy diet, protein sparing modified fasting

## Abstract

**Introduction:**

The Very Low-Calorie Ketogenic Diet (VLCKD) has emerged as a safe and effective intervention for the management of metabolic disease. Studies examining weight loss predictors are scarce and none has investigated such factors upon VLCKD treatment. Among the molecules involved in energy homeostasis and, more specifically, in metabolic changes induced by ketogenic diets, Fibroblast Growth Factor 21 (FGF21) is a hepatokine with physiology that is still unclear.

**Methods:**

We evaluated the impact of a VLCKD on weight loss and metabolic parameters and assessed weight loss predictors, including FGF21. VLCKD is a severely restricted diet (<800 Kcal/die), characterized by a very low carbohydrate intake (<50 g/day), 1.2–1.5 g protein/kg of ideal body weight and 15–30 g of fat/day. We treated 34 patients with obesity with a VLCKD for 45 days. Anthropometric parameters, body composition, and blood and urine chemistry were measured before and after treatment.

**Results:**

We found a significant improvement in body weight and composition and most metabolic parameters. Circulating FGF21 decreased significantly after the VLCKD [194.0 (137.6–284.6) to 167.8 (90.9–281.5) *p* < 0.001] and greater weight loss was predicted by lower baseline FGF21 (Beta = −0.410; *p* = 0.012), male sex (Beta = 0.472; *p* = 0.011), and central obesity (Beta = 0.481; *p* = 0.005).

**Discussion:**

VLCKD is a safe and effective treatment for obesity and obesity related metabolic derangements. Men with central obesity and lower circulating FGF21 may benefit more than others in terms of weight loss obtained following this diet. Further studies investigating whether this is specific to this diet or to any caloric restriction are warranted.

## 1. Introduction

In the last decades there has been a growing interest for the Very Low-Calorie Ketogenic Diet (VLCKD) as a feasible nutritional intervention for the management of obesity providing significant weight loss and improvement in obesity-related diseases. This diet is characterized by a very low carbohydrate intake (< 50 g/day), 1.2–1.5 g protein/kg of ideal body weight, 15–30 g of fat/day, in the context of a very low-calorie intake (approximately 800 kcal/day) (1). The restriction of carbohydrates induces the lipolysis of fat depots and leads to nutritional ketosis, modulating the gut microbiome (2) and inducing a metabolic effect that stabilizes glucose levels and minimizes insulin release (3). Circulating levels of ketone bodies, especially B-hydroxybutyrate (BHB), promote an anorexigenic effect, reducing appetite and food intake (4, 5), which is one of the mechanisms accounting for the tolerability and high adherence to such a restrictive diet (6). Recently, a meta-analysis was conducted to assess the efficacy of the VLCKD in subjects with overweight and obesity (7). The main results reported a significant weight loss in the short, intermediate, and long term and improvements in body composition parameters (reducing waist circumference and fat mass without inducing lean mass loss) as well as glucose and lipid profile (8). In particular, the VLCKD is associated with a larger reduction in fasting glucose, the homeostasis model of assessment-IR (HOMA-IR) index, total cholesterol and triglycerides levels, compared to other weight loss programs such as balanced low-calorie diets (7). Emerging evidence suggests that ketogenic diets may have several applications in the treatment of metabolic disease, including NAFLD and type 2 diabetes (9–11), as well as pre (12) and post bariatric surgery (13). More generally speaking, carbohydrate restriction even in the form of high fat ketogenic diets was proven to be anti-inflammatory and effective in improving several metabolic parameters compared to control (14–16), suggesting that a low carbohydrate approach may be more beneficial compared to isocaloric diets with higher carbohydrate content. However, it should be pointed out that evidence on this regard is still limited, and further studies comparing strictly isocaloric diets in a residential setting should be conducted in order to confirm this. Interestingly, emerging evidence also suggests that exogenous ketone bodies may prove beneficial, but more studies are needed to further elucidate their effects (17). However, the extent of weight loss with VLCKD highly varies among patients and some factors, including the presence of specific genetic variants, have been supposed to cause this variability (18). Beside this evidence, while the metabolic predictors of weight reduction were investigated for other lifestyle therapeutic approaches (19, 20) no study investigated metabolic predictors of weight loss after VLCKD treatment.

Among the many molecules involved in energy homeostasis and, more specifically, in metabolic changes induced by ketogenic diets, Fibroblast Growth Factor 21 (FGF21) is a hormone predominantly secreted by the liver that exerts endocrine and paracrine effects, although its physiology is not fully understood yet (21, 22). The association between FGF21 and obesity in humans seems more complex and controversial than that observed in primate and murine models (23). Mice fed a ketogenic diet express increased FGF21 levels (24, 25), whereas our group and others have demonstrated that ketogenic diets result in decreased FGF21 levels in human subjects (26, 27). Moreover, the positive correlation with obesity, insulin resistance (IR) and metabolic syndrome (MetsS) has been confirmed *in vivo* (21), which, together with the known *in vitro* effects of this hepatokine, makes it reasonable to hypothesize that FGF21 may play a role in the response to weight loss interventions.

With the prevalence of obesity steadily increasing in most countries (28, 29), it is of utmost importance to find effective treatments, and nutrition strategy personalization is key. Identifying predictors of weight loss prior to specific treatment initiation may help choosing the right treatment for each patient, likely enhancing the success rate. In fact, the first cause of diet discontinuation is the poor response to the diet (30). In addition, recognizing weight loss predictors among novel molecules may help generating hypotheses regarding the physiology of weight loss, possibly paving the way for further mechanistic studies.

In this preliminary report, we aimed to assess baseline predictors of greater weight loss in patients with obesity undergoing a VLCKD focusing on the predictive role of FGF21.

## 2. Materials and methods

### 2.1. Study design and population

In this single-center, observational prospective before-after study, we evaluated baseline predictors of weight loss amount after 45 VLCKD diet. Variables collected at baseline were: demographic data, anthropometric data, glycol-metabolic data, liver function tests, kidney function test, C-reactive protein and FGF21.

The primary study outcome was to investigate the predictive role of FGF21 on weight loss amount after short-term VLCKD diet. Secondary outcomes were to investigate which of the routinary clinical, anthropometrical, or biochemical baseline characteristics predict a larger weight loss in these patients.

Weight loss amount was defined as before-after weight delta.

Patients were enrolled in the Center of High Specialization for the Care of Obesity (CASCO), Rome, Italy. The inclusion criteria were: BMI over 30 kg/m^2^, 18–60 years of age; stable body weight in the preceding 3 months. No gender ratio was set upon enrolment. Exclusion criteria were: contraindications to a VLCKD, as severe organ failure, insulin dependent diabetes, current pregnancy or breastfeeding, any allergy to meal replacements components impossible to be avoided, no signed informed consent, psychiatric diseases possibly hindering compliance (31).

### 2.2. Intervention

All patients followed a VLCKD with meal replacements (New Penta s.r.l., Cuneo, Italy) for 45 days at home. Participants were clinically evaluated at baseline and every 2 weeks up to the end of the study. All patients could contact the dieticians directly by phone or sms whenever needed in order to improve adherence. They were encouraged to reduce their sedentary lifestyle, although no formal exercise program was provided. The nutritional intervention was ∼800 kcal/day, consisting of 4 or 5 meal replacements daily which were provided to the patient at each follow up visit, and one serving of vegetables with a low glycemic index at lunch and dinner, which the patients were required to purchase and prepare autonomously at home. The composition was as follows: carbohydrates 26 g, protein 1.2–1.5 g/Kg of ideal body weight (32), fat 35 g. The protein source in the meal replacements mainly came from whey, egg, and soy, and fats were from extra virgin olive oil. All patients were encouraged to drink at least 2 L of water daily and to take daily multimineral and vitamin supplements which were provided as per current recommendations (7). The patients were also provided with urine test strips for acetoacetate and were asked to self-test the first morning urine weekly. The same strips were used at each visit to confirm compliance to the VLCKD.

### 2.3. Measurements

All subjects were evaluated before and right after the end of the dietary intervention. The same stadiometer and calibrated scale were used to measure height and body weight. Waist circumference (WC) was measured midway between the lower rib and the iliac crest, at the end of a normal expiration. Hip circumference (HC) was measured at the level of the widest circumference over the great trochanters to the closest 1.0 cm. The waist-to-hip (W/H) ratio was calculated as WC divided by HC. An automated device was used to measure at each visit.

### 2.4. Body composition evaluation

All subjects had their body composition assessed before and right after the end of the dietary intervention through dual-energy-X-ray absorptiometry (DXA) (Hologic 4500, Bedford, MA, USA) as previously described (33).

### 2.5. Laboratory assay

All patients’ blood samples were drawn in the morning following an overnight fast at baseline and at the end of the treatment. The parameters measured at the hospital laboratory following the local standards of practice were complete blood count, total, HDL and LDL cholesterol, triglycerides, electrolytes, glucose and insulin, albumin, C-reactive protein (CRP), creatinine and estimated glomerular filtration rate (eGFR), alanine transaminase (ALT), aspartate transaminase (AST), uric acid. FGF21 serum levels were measured after an overnight fast using a commercial assay (R&D Systems, Inc., Minneapolis, MN, USA). Metabolic Syndrome (MetS) was defined by the modified ATP-III criteria (34). Urinary acetoacetate was self-measured in the first morning urine at baseline and weekly until the end of the study (Ketur-Test, Accu-Chek, Roche Diagnostics, Rome, Italy), to monitor dietary adherence. Patient reporting negative urinary tests more than once were to be excluded. IR was determined through HOMA-IR calculation as (35):


$$\text{HOMA}-\text{IR}=\frac{\begin{array}{c} \mathrm{fasting}\,\mathrm{serum}\,\mathrm{insulin}\,\left(\text{mIU}/\text{ml}\right)\\ \times \mathrm{fasting}\,\mathrm{plasma}\,\mathrm{glucose}\,\left(\text{mg}/\text{dL}\right) \end{array}}{405}$$

### 2.6. Statistical analysis

Data are expressed as mean and standard deviation for normally distributed variables and median and interquartile range for non-normally distributed ones. Group comparisons were performed by unpaired Student’s *t*-test and ANOVA test or by Mann–Whitney and Kruskal–Wallis test as appropriate. Proportions and categorical variables were tested by the Chi square test. Data before and after intervention were compared with paired *t*-test or Wilcoxon rank test. A Spearman correlation method was used to analyze the correlation between continuous variables. To test independent predictors of weight loss, a first multivariate analysis was performed including demographic variables (gender and age) as clinically relevant factors and variable significantly correlated to weight loss at univariate analysis (baseline weight, W/H ratio, FGF21), after excluding collinear variables (lean mass, waist circumference). Non-normally distributed variables were log-transformed. Further multivariate analyses were performed including either sex or W/H ratio. Analyses were performed using computer software packages (SPSS-27.0, SPSS Inc., Armonk, NY, USA: IBM Corp.).

The mean ± SD body weight we observed in the population accessing our clinical center was 105 ± 21. Twenty-nine patients were identified as an appropriate sample size to detect a clinically relevant reduction of 10% in body weight with a power of 0.80 and alpha 0.05. Foreseeing up to 20% drop-out rate, 34 patients were then enrolled.

### 2.7. Ethical approval

The study was carried out in accordance with the code of ethics of the World Medical Association for human studies (Declaration of Helsinki, 2001). All patients signed an informed consent form to voluntarily participate in this study. The research protocol was approved by the Ethical Committee of Sapienza University of Rome (rif. 5475, date of approval 24-10-2019).

## 3. Results

### 3.1. Anthropometric and biochemical changes

A total of 34 patients [14 male (41%), 20 female (59%)] were enrolled in this study. The mean age was 54 ± 12 years, the mean BMI was 36.3 ± 4.1 kg/m^2^. The baseline characteristics of our population are reported together with variations after VCLKD treatment in Table 1. All patients had detectable urinary acetoacetate reflecting ketosis until the end of the diet. No patient dropped out during the study due to extreme hunger or intolerable physical symptoms. No significant adverse event was recorded. The most common minor adverse events recorded were bloating, constipation, headache and self-limiting palpitations. All symptoms were deemed as bearable by all patients and most were controlled by increasing water intake or adjusting the quality or quantity of consumed vegetables. All patients reported an improvement in their sedentary lifestyle, however no one started light or moderate-intensity aerobic physical activity during the VLCKD treatment.

**TABLE 1:** Participants characteristics at baseline (T0), and after the very low-calorie ketogenic diet (VLCKD) (T45).

	T0 *n* = 34	T45 *n* = 34	*p*
Age (years)	54 ± 12	54 ± 12	
Gender (% female)	59	59	
Weight (Kg)	102.144 ± 13.1	93.8 ± 13.3	<0.001
BMI (kg/m^2^)	36.3 ± 4.1	33.29 ± 4.0	<0.001
WC (cm)	108.6 ± 8.9	102.2 ± 8.8	< 0.001
W/H ratio	0.8 ± 0.1	0.9 ± 0.1	< 0.001
FGF21 (ng/ml)	194.0 (137.6–284.6)	167.8 (90.9–281.5)	<0.001
Glucose (mg/dL)	102.5 (94–108.5)	95.5 (88–106)	0.004
Insulin (μUI/ml)	17 (13.4–22.6)	7 (5.12–12.5)	<0.001
HOMA IR	4.3 (3.1–5.9)	1.9 (1.1–3.2)	< 0.001
Triglycerides (mg/dL)	133.3 ± 53.9	96.9 ± 45.9	<0.001
Total cholesterol (mg/dL)	215.74 ± 36.9	177.1 ± 37.1	<0.001
LDL cholesterol (mg/dL)	137.1 ± 32.8	103.7 ± 36.3	<0.001
HDL cholesterol (mg/dL)	52.1 ± 13.6	49.4 ± 12.4	0.112
Albumin (g/dL)	44.3 ± 2.6	43.9 ± 2.8	0.350
AST (U/L)	20 (16–23)	19.5 (17–23)	0.903
ALT (U/L)	23.5 (18–35.5)	20.5 (18–30.75)	0.174
Creatinine (mg/dL)	0.8 (0.7–0.9)	0.8 (0.7–0.9)	0.969
CRP (μg/dL)	3200 (1900–8100)	2750 (905–5900)	0.340
Lean Mass (kg)	61.03 ± 9.4	59.3 ± 9.7	0.058
Fat Mass (kg)	38.0 ± 9.1	33.3 ± 8.8	<0.001
Fat mass (%)	37.1 ± 7.3	34.9 ± 7.6	<0.001

Variables with normal distribution are expressed as mean ± SD, those with non-normal distribution as median (interquartile range). BMI, body mass index; WC, waist circumference; W/H ratio, waist-to-hip circumference ratio; FGF21, fibroblast growth factor 21; HOMA-IR, homeostasis model assessment-insulin resistance; AST, aspartate transaminase; ALT, alanine transaminase; CRP, C-reactive protein. *p* is from a mixed-effects analysis.

Body weight (102.1 kg ± 13.1 to 93.8 kg ± 13.3, *p* < 0.001), BMI (36.3 ± 4.1 kg/m^2^ to 33.3 kg/m^2^ ± 4.0, *p* < 0.001), WC (108.6 cm ± 8.9 to 102.2 cm ± 8.8, *p* < 0.001), and W/H ratio (0.8 ± 0.1 to 0.9 ± 0.1, *p* < 0.001) were significantly improved at the end of the diet (Table 1). Regarding body composition, the VLCKD induced a significant decrease in fat mass (38.0 kg ± 9.1 to 33.3 kg ± 8.8, *p* < *0.001*), and fat mass percentage (37.1% ± 7.3 to 34.9% ± 7.6, *p* < *0.001*), No difference was observed in absolute lean mass.

Metabolic parameters achieved a significant change, including fasting glucose [102.5 mg/dL (94.0–108.5) to 95.5 mg/dL (88.0–106.0), *p* < 0.004] and insulin [17.0 μUI/ml (13.4–22.6) to 7.0 μUI/ml (5.1–12.5), *p* < 0.001], triglycerides (133.3 mg/dL ± 53.9 to 96.9 mg/dL ± 45.9, *p* < 0.001), total cholesterol (215.7 mg/dL ± 36.9 to 177.1 mg/dL ± 37.1, *p* < 0.001), LDL cholesterol (137.1 mg/dL ± 32.8 to 103.7 mg/dL ± 36.3 *p* < 0.001), and HOMA-IR [4.3 (3.1–5.9) to 1.0 (1.1–3.2), *p* < 0.001]. No differences were observed in AST, ALT, creatinine or in albumin levels. Circulating FGF21 level decreased significantly from baseline to the end of the treatment [194.0 ng/ml (137.6–284.6) to 167.8 ng/ml (90.9–281.5) *p* < 0.001]. CRP was unchanged.

### 3.2. Predictors of weight loss

In order to assess whether parameters included in the evaluation conducted at baseline could predict the weight loss obtained with a VLCKD, the study population was stratified according to weight loss tertiles, reported in kg, after VLCKD treatment. Baseline metabolic markers, anthropometric parameters and body composition are reported according to weight loss tertiles in Table 2. Patients in the highest weight loss tertile showed higher baseline values of W/H ratio and MetS prevalence; this sub-group of patients also showed lower baseline circulating FGF21 levels. No significant difference among groups was observed regarding baseline BMI, BW, WC, albumin, glucose, insulin, HOMA-IR, AST, ALT, CRP, lean mass, and fat mass.

**TABLE 2:** Baseline clinical, biochemical characteristics and body composition of patients according to weight loss, stratified into tertiles.

	I tertile (*n* = 11)	II tertile (*n* = 12)	III tertile (*n* = 11)	*P* among groups	*P* I vs. III tertile
Age (years)	52.7 ± 5.7	55.6 ± 7.9	55.6 ± 10.1	0.630	1.00
Female sex	72.7%	50.0%	54.5%	0.510	
Weight (kg)	101.9 ± 7.1	105.1 ± 14.6	99.2 ± 16.3	0.572	1.00
BMI (kg/m^2^)	36.7 ± 4.6	37.1 ± 4.3	35.1 ± 3.7	0.507	1.00
WC (cm)	106.3 ± 9.3	111.2 ± 9.9	108.2 ± 7.5	0.419	1.00
W/h ratio	0.8 ± 0.1	0.9 ± 0.1	0.9 ± 0.1	0.092	0.094
MetS	63.6%	75.0%	100.0%	0.097	0.027
WC	100.0%	100.0%	100.0%	–	–
Hypertension	72.7%	100.0%	100.0%	0.032	0.062
Hyperglycaemia	54.5%	58.3%	81.8%	0.346	0.170
HyperTG	27.3%	25.0%	45.5%	0.525	0.375
Low HDL-C	27.3%	41.7%	81.8%	0.029	0.010
FGF21 (ng/ml)	246.5 (140.6–315.3)	214.5 (180.64–367.7)	148.5 (70.7–196.2)	0.038	0.045
Glucose (mg/dL)	101.0 (88.0–108.0)	99 (94.75–109.5)	103 (94–117)	0.794	0.532
Insulin (μUI/ml)	17.4 (12.3–26.3)	17.8 (14.7–19.8)	15.1 (13.2–27.6)	0.784	0.870
HOMA-IR	5.4 (2.6–6.8)	4.4 (3.8–5.5)	3.8 (2.9–6.9)	0.963	0.974
Albumin (g/dL)	43.8 ± 2.6	43.9 ± 2.7	45.3 ± 2.4	0.324	0.586
AST (U/L)	22 (14–25)	19 (15.2–23.7)	20.0 (17–22)	0.460	0.154
ALT (U/L)	27 (21–42)	21.5 (17.5–36.5)	22 (18–31)	0.456	0.307
CRP (mg/dL)	4.4 (2.2–11)	3.3 (2.1–7.7)	2.5 (0.7–3.7)	0.154	0.071
Lean Mass (kg)	59.7 ± 8.5	60.3 ± 7.2	63.3 ± 12.3	0.637	1.000
Fat Mass (kg)	40.1 ± 8.0	40.1 ± 8.5	33.7 ± 10.1	0.165	0.306

BMI, body mass index; WC, waist circumference; W/h ratio, waist circumference/Hip circumference ratio; Mets, metabolic syndrome; HDL-C, HDL cholesterol; FGF21, fibroblast growth factor 21; HOMA-IR, homeostasis model assessment-insulin resistance; AST, aspartate transaminase; ALT, alanine transaminase; CRP, C-reactive protein.

**FIGURE 1:**
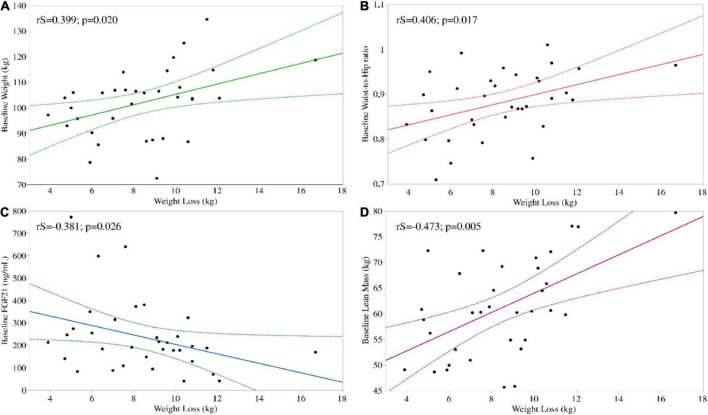
Significant univariate associations between baseline parameters and weight loss following a VLCKD. Weight loss correlated positively with baseline body weight **(A)** and W/H ratio **(B)** and negatively with baseline circulating FGF21 **(C)**. Regarding body composition, weight loss positively correlated with baseline lean mass **(D).**

To further investigate whether weight loss was associated to any collected baseline parameter, so to identify possible predictors of weight loss following a VLCKD, we performed a univariate analysis. Weight loss positively correlated with baseline body weight (*r*S = 0.399; *p* = 0.020), waist circumference (*r*S = 0.359; *p* = 0.037), W/H ratio (*r*S = 0.406; *p* = 0.017) and was negatively correlated with baseline circulating FGF21 levels (*r*S = –0.381; *p* = 0.026). Regarding body composition, weight loss positively correlated with baseline lean mass (*r*S = 0.473; *p* = 0.005) (Figure 1) but not with baseline fat mass (*r*S = –0.132; *p* = 0.456) or HOMA-IR (*r*S = 0.129; 0.466).

We therefore proceeded to test independent predictors of weight loss. To do so, we included in a first multivariate analysis demographic variables (gender and age) as clinically relevant factors and variables which were found to be significantly correlated to weight loss at univariate analysis (baseline weight, W/H ratio, FGF21). After correction for age, sex, W/H ratio and baseline weight, weight loss correlated with FGF21 (Beta = –0.410; *p* = 0.012) (Table 3, panel A). In further analyses, including alternatively sex (Table 3, panel B), or W/H ratio (Table 3, panel C) we observed that, in addition to FGF21, male sex (Beta = 0.472; *p* = 0.001) and W/H ratio (Beta = 0.481; *p* = 0.005) correlated with weight loss as well. No difference in FGF21 level was found between male and female.

**TABLE 3:** Multiple regression analysis to assess baseline predictors of body weight loss.

Panel A	B	S.E.	Beta	*p*
Age	–4.43E-5	0.003	-0.002	0.987
Male sex	0.066	0.064	0.230	0.316
Baseline W/H ratio	0.675	0.414	0.340	0.115
Baseline body Weight (kg)	0.002	0.002	0.171	0.336
Baseline FGF21^#^	–0.199	0.075	-0.410	0.012
**Panel B**	**B**	**S.E.**	**Beta**	***p***
Age	0.001	0.003	0.060	0.700
Male sex	0.135	0.050	0.472	0.011
Baseline body Weight (kg)	0.001	0.002	0.099	0.573
Baseline FGF21^#^	–0.198	0.077	-0.407	0.015
**Panel C**	**B**	**S.E.**	**Beta**	***p***
Age (years)	0.000	0.003	0.007	0.962
Baseline W/H ratio	0.954	0.311	0.481	0.005
Baseline body Weight (kg)	0.003	0.002	0.266	0.336
Baseline FGF21^#^	–0.183	0.073	-0.376	0.018

Independent variables evaluated: age, male sex, baseline body weight, baseline FGF21, baseline waist-to-hip circumference ratio (W/H ratio) (panel A). Panel (B) reports the model after the removal of W/H ratio. Panel (C) reports the model after the removal of the sex variable. SE, standard error. ^#^Log transformed variables.

## 4. Discussion

We report a significant reduction of fat mass and weight with improvement of metabolic parameters in patients with obesity following a VLCKD for 45 days, with no dropouts or significant adverse events. These results are in line with previous studies reporting that the VLCKD is both safe and effective in subjects with obesity, since it promotes satiety, rapid weight loss preserving lean mass and metabolic improvement (7, 36, 37). The extremely high adherence was likely due to the fact that all patients could contact their dietician directly by phone or sms whenever needed. Remote monitoring is a well-established means of achieving good compliance, especially in nutritional studies (38).

A higher W/H ratio predicted greater weight loss in this population, in line with previous studies (39). Central obesity, reflecting increased visceral fat, is typically unhealthy, and it comes with increased cardiovascular disease risk and metabolic derangements. It was previously reported that visceral fat depots retain higher lipolytic activity compared to peripheral depots (40), and that diet-induced weight loss is associated with a greater visceral fat loss compared to peripheral fat loss (41). This may explain the finding that those with a prevalence of fat more likely to be decreased upon dieting will obtain more profound weight loss. Moreover, the gut microbiome of the metabolically unhealthy individual has a specific signature, and it was previously reported that this represents a significant predictor of weight loss upon dieting, with specific gut bacteria likely synergizing with or counteracting macronutrients to influence weight loss (42). We previously reported that a VLCKD modulates the microbiome toward a healthier phenotype (43). We therefore suggest that under the role of W/H ratio as predictor of weight loss may also lie a fine interaction between dietary factors and the gut microbiome in determining the weight loss trajectory, but further studies are needed to confirm this.

We found that male gender predicted greater weight loss. As men have a larger amount of central fat, we cannot exclude that the finding may be pulled by this aspect, with the previously mentioned implications. In addition, men also have higher lean mass. Organs and tissues that constitute lean mass have high metabolic activity, accounting for ∼70% of the variance in resting energy expenditure (44), making it the most important determinant of energy expenditure in sedentary subjects with obesity. As the caloric intake prescribed was similar across patients, it accounted for a more profound energy deficit in those with higher energy expenditure (i.e., men), likely leading to larger weight loss. Confirming this, greater weight loss was observed in patients with higher lean mass at baseline. The finding was therefore expected and in line with previous studies (45). Noteworthy, studies investigating predictors of weight loss following bariatric surgery or pharmacologic treatment showed contrasting data, with some identifying male sex as a negative predictor of weight loss (46, 47), and others suggesting that males lose more weight (48). This is likely due to the heterogeneity in the cohorts and treatments evaluated, and no definitive conclusion may be drawn in this regard.

We report decreased circulating FGF21 after a VLCKD intervention, in line with previous studies, and that lower baseline FGF21 predicts greater weight loss even after adjustment for age, sex and baseline body weight. In humans, circulating FGF21 levels are regulated by different conditions, from the fasting to the refed state (49, 50), high carbohydrate and fructose consumption (51, 52), or dietary protein restriction (53). However, the mechanism underlying these effects is not entirely clear (54). Furthermore, higher FGF21 level was detected in different metabolic disorders, including type 2 diabetes (55), obesity and liver steatosis (56, 57), pancreatitis (58), and primary mitochondrial dysfunction (59). In a previous study, Crujeiras et al. proved a significant reduction in FGF21 in patients who lost weight using different diet types, including VLCKD. Conversely, they found increased levels in patients who underwent bariatric surgery, suggesting that FGF21 could represent a nutritional stress marker (60). The reason why patients losing more weight presented lower baseline FGF21 is unclear, but an explanation may be hypothesized. It has been reported by Fisher et al. for the first time, that obesity may be an FGF21-resistant state (61). Similar to the mechanism of insulin resistance (62), patients with lower FGF21 levels, possibly reflecting lower FGF21 resistance, could lose weight more easily as a consequence of a healthier and more flexible metabolism. However, in our study the extent of weight loss inversely correlates with FGF21 but not with HOMA-IR. HOMA-IR is a widely accepted marker of insulin resistance and it is influenced by the metabolic status of adipocytes (63), liver (64), and muscle cells (65). Differently, FGF21 mainly reflects liver metabolism (21, 22). Based on these differences, we could speculate that the residual liver function could play a key role in the extent of the weight loss following VLCKD, more than the levels of adipokines secretion or the metabolic status of muscle cells. However, further studies comparing the response to VLCKD of patients with or without hepatic disorders or with different stage of liver disease are needed to test our hypothesis.

In addition, ketogenic diets exert immunomodulatory actions (2, 66, 67). Chronic low-grade inflammation is the culprit of metabolically unhealthy obesity (68), and FGF21 is secreted by the liver upon inflammatory *stimuli* (69, 70). The changes observed upon VLCKD treatment in terms of FGF21, as well as the possibly weight loss predictive role of baseline FGF21, may not currently have clinical implications, but they suggest that FGF21 could represent a link in the chain connecting the metabolic and immune system, although further mechanistic studies are needed to elucidate this.

Our study has some limitations. First, the absence of a control group following an isocaloric dietary intervention with different macronutrient composition did not allow to investigate the effect of the macronutrient ratio/nutritional ketosis as opposed to calorie restriction derived weight loss. The results observed are therefore the consequence of the dietary treatment as a whole, without speculations possibly being made on whether it was the very low-calorie content or the nutritional ketosis achieved to impact the weight or other metabolic parameters. Although a control group would have allowed to assess whether different diets have the same predictors of weight loss, this was beyond the scope of the present study, and we aim at further pursuing this important aspect. Second, capillary BHB was not measured to confirm ketosis, although less reliable urinary acetoacetate confirmed ketonuria throughout the study in all subjects. Third, the sample size enrolled in the study was relatively small. However, *a priori* sample size was calculated allowing for sufficient power. Fourth, the duration was short, and no long term follow up was conducted. No direct evaluation of energy expenditure was adopted, neither was a validated questionnaire to monitor physical activity administered. It was therefore not possible to accurately calculate total daily energy expenditure, and the prescribed energy deficit was hence different across patients, possibly accounting for some bias. Finally, no basal metabolic rate or respiratory quotient were measured in our patients, not allowing the evaluation of the impact of energy delta between diet calorie consumption and expenditure upon weight loss.

## 5. Conclusion

In conclusion, men with central obesity and lower circulating FGF21 may benefit more than others in terms of weight loss obtained following this diet. Further controlled, larger and longer studies investigating whether this is specific to the VCLKD or to any caloric restriction need to be conducted before any definitive conclusion may be drawn.

## Data availability statement

The raw data supporting the conclusions of this article will be made available by the authors, without undue reservation.

## Ethics statement

The studies involving human participants were reviewed and approved by the Ethical Committee of Sapienza University of Rome (rif. 5475, date of approval 24-10-2019). The patients/participants provided their written informed consent to participate in this study.

## Author contributions

SB and FB: conceptualization. SB, GS, and LG: methodology. RR, MW, AP, IE, and DT: software. MW, SB, LG, AG, and CL: validation. IE, FB, and MW: formal analysis. SB, AP, SM, and EC: investigation. AP and RR: data curation. MW, IE, and DT: writing—original draft preparation. MW, LG, SB, SM, and CL: writing—review and editing. LG: supervision. SB: project administration. SB, GS, LG, and MW: funding acquisition. All authors have read and agreed to the published version of the manuscript.
